# Complete Genome Sequence of Escherichia coli J53, an Azide-Resistant Laboratory Strain Used for Conjugation Experiments

**DOI:** 10.1128/genomeA.00433-18

**Published:** 2018-05-24

**Authors:** Yasufumi Matsumura, Gisele Peirano, Johann D. D. Pitout

**Affiliations:** a Department of Clinical Laboratory Medicine, Kyoto University Graduate School of Medicine, Kyoto, Japan; b Departments of Pathology & Laboratory Medicine, University of Calgary, Calgary, Alberta, Canada; c Division of Microbiology, Calgary Laboratory Services, Calgary, Alberta, Canada; d Departments of Microbiology, Immunology and Infectious Diseases, University of Calgary, Calgary, Alberta, Canada; e Department of Medical Microbiology, University of Pretoria, Pretoria, South Africa

## Abstract

We report here the complete genome sequence of Escherichia coli J53, which is used as a recipient in conjugation experiments and is a laboratory strain derived from E. coli K-12. This genome sequence will help in the development of a comprehensive genetic analysis of conjugative elements.

## GENOME ANNOUNCEMENT

Escherichia coli J53 is a laboratory mutant of E. coli K-12 (1). This strain is negative for fertility factors and is resistant to sodium azide. It has been used for conjugation experiments as a recipient strain. Recent advances in sequencing technology have enabled the sequencing of transconjugants, which contribute to a comprehensive understanding of transferable elements. The complete genome sequence of J53 is essential for accurate genomic analysis, but currently, there is only one draft genome assembly available (GenBank accession no. AICK00000000, hereinafter referred to as J53_AICK) (2). Here, we present a complete genome sequence for E. coli J53, an isolate that has been used for conjugation experiments in Calgary, Alberta, Canada (3).

The whole-genome DNA was sequenced using both Illumina NextSeq 500 (150-bp paired-end) and Oxford Nanopore Technologies MinION (R9.4 flow cell) systems. A circular chromosome was obtained by a *de novo* hybrid assembly pipeline of Unicycler version 0.4.4 (4). The assembly presented an average coverage of 188× by NextSeq 500 and of 1,530× by MinION. The genome was annotated using the NCBI Prokaryotic Genome Annotation Pipeline (5).

The complete genome contains a 4,682,574-bp chromosome with a GC content of 50.8%, 4,530 coding sequences, 82 tRNA-coding genes, and 22 rRNA-coding operons. The strain belongs to sequence type 10, as per the Achtman multilocus sequence typing scheme (http://mlst.warwick.ac.uk/mlst/dbs/Ecoli), similar to other E. coli K-12 strains. A genomic comparison against the K-12 reference genome for substrain MG1655 (GenBank accession no. U00096) using progressiveMauve (6) revealed that J53 had a total of 17,012 bp of deletions, including IS*1A* (2 copies), IS*5*, *proB*, *proA*, four tRNAs, a part of prophage CP4-6, and a part of a repetitive extragenic palindromic sequence (REP321) in 7 segments. A total of 57,947 bp of insertions included IS*10R* (6 copies), rtT small RNA (sRNA), and the lambda prophage (GenBank accession no. J02459), in 8 segments. J53 contained an 825,667-bp inversion block flanked by 2 copies of IS*5* between nucleotide positions 3652638 and 4476957 of the MG1655 genome.

Using the 39 E. coli K-12 genomes available on the NCBI Assembly resource (https://www.ncbi.nlm.nih.gov/assembly/) as of 9 April 2018 and the J53_AICK draft genome sequence, we made a phylogenetic tree based on core single nucleotide polymorphisms (SNPs) with kSNP3.0 (7). The J53 and J53_AICK genomes formed a cluster which was different from a cluster made up of 15 MG1655 genomes. Between the J53 and J53_AICK genomes, 58 SNPs were present. They had different SNPs in the *secA* gene, which is associated with azide resistance (8), resulting in different amino acid alterations (F598L in J53 and A112V in J53_AICK). J53 had a total of 63,175 bp of insertions and a total of 28,749 bp of deletions compared with J53_AICK. These differences in the core and accessory genomes suggest that the J53 strain from our collection and the J53_AICK from South Korea have a common ancestor but have acquired mutations during serial passages. This finding highlights the importance of sequencing strains that are utilized for genomic experiments.

### Accession number(s).

The complete sequence of the chromosome of E. coli J53 has been deposited in GenBank under accession no. CP028702.
